# Cold-spot driven local failure after stereotactic body radiation therapy for colorectal liver metastases

**DOI:** 10.3389/fonc.2026.1800905

**Published:** 2026-04-17

**Authors:** Hyunju Shin, Jeong Il Yu, Hee Chul Park, Sang Hoon Seo, Seong Hyeon Yun, Woo Yong Lee, Hee Cheol Kim, Yong Beom Cho, Yoon Ah Park, Jung Wook Huh, Jung Kyong Shin, Young Suk Park, Jeeyun Lee, Seung Tae Kim, Sung Hee Lim, Ji Eun Shin, Nalee Kim

**Affiliations:** 1 Department of Radiation Oncology, Samsung Medical Center, Sungkyunkwan University School of Medicine, Seoul, Republic of Korea; 2 Department of Surgery, Samsung Medical Center, Sungkyunkwan University School of Medicine, Seoul, Republic of Korea; 3 Division of Hematology-Oncology, Department of Medicine, Samsung Medical Center, Sungkyunkwan University School of Medicine, Seoul, Republic of Korea

**Keywords:** colorectal cancer, dosimetric parameter, liver metastasis, plan quality, radiation therapy

## Abstract

**Background:**

This single-institution retrospective study investigated whether actual dosimetric plan quality metrics better predict freedom from local progression (FFLP) than nominal prescription dose after stereotactic body radiation therapy (SBRT) for colorectal liver metastases (CLM).

**Methods:**

A total of 116 patients with 128 CLM treated with SBRT (≥70 Gy biologically effective dose [BED_10_]) were retrospectively analyzed. Clinical variables and dosimetric factors, including maximum, minimum, and mean doses (Dmax, Dmin, Dmean), as well as dose to x% of the planning target volume (PTV) (Dx%), were evaluated. Cox regression analyses and the Akaike information criterion were used to identify prognostic factors for FFLP.

**Results:**

With a median follow-up of 22.3 months, 1-year and 2-year FFLP rates were 74.5% and 58.8%, respectively. On univariable analyses, extent of metastasis, number of systemic therapy lines, pre- and post-SBRT carcinoembryonic antigen, PTV, prescription dose, and other plan parameters (Dmax, Dmin, Dmean, D2%, D95%, D98%, D99%) were significant. In the final multivariable model, PTV Dmin (HR 0.89 per 10 Gy BED_10_, 95% CI 0.83–0.96, p = 0.002) and polymetastatic disease (HR 3.31, 95% CI 1.79–6.12, p < 0.001) independently predicted FFLP. Using an optimal cut-point of 100 Gy BED_10_, low-PTV Dmin was associated with inferior FFLP compared with high-PTV Dmin (1-year: 64.2% vs. 87.1%, p < 0.001).

**Conclusion:**

PTV Dmin emerged as the most robust dosimetric predictor of FFLP. Incorporating PTV Dmin into SBRT planning may improve FFLP beyond nominal prescription dose.

## Introduction

The paradigm of oligometastasis has increased clinical interest in aggressive local therapies for metastatic cancer (1–3). For colorectal liver metastasis (CLM), while surgical resection and radiofrequency ablation remain the main modalities, their feasibility is often limited by unfavorable anatomical locations, tumor size, insufficient liver remnant volume, or comorbidities. In such scenarios, stereotactic body radiation therapy (SBRT) serves as a viable and effective alternative (3–6). The therapeutic effect of SBRT is dose-dependent, but even with identical prescription doses, actual dose delivery can vary considerably due to anatomical differences, respiratory motion, and organ-at-risk constraints (7, 8). Specific plan quality metrics might more accurately represent delivered dose heterogeneity and may explain prognostic differences not captured by prescription dose alone. However, most previous studies have primarily focused on prescribed dose, and data on the impact of actual plan quality on clinical outcomes in CLM remain limited (9–11).

Therefore, the present study aims to quantitatively assess the association between dosimetric metrics and freedom from local progression (FFLP) in patients receiving SBRT for CLM. By evaluating whether actual plan quality more accurately predicts therapeutic outcomes than prescription dose alone, this study proposes to establish objective and clinically relevant benchmarks to guide future RT planning and clinical decision-making.

## Methods

### Study design and patients

We retrospectively reviewed medical records of patients who received SBRT for CLM between May 2016 and June 2024. In this study, SBRT was defined as high-precision ablative radiation therapy (RT) (≥5 GyE per fraction, ≤15 fractions), and only cases with a ≥ 70 Gy biologically effective dose (BED) with α/β of 10 were included. Patients were excluded if planned SBRT was not completed, and if the follow-up period was less than 3 months. Oligometastasis was defined as the presence of up to five metastatic lesions at the time of SBRT. Disease status was also classified according to the consensus recommendation of characterization and classification of oligometastatic disease by the European Society for Radiotherapy and Oncology (ESTRO) and European Organization for Research and Treatment of Cancer (EORTC) (12). Baseline liver function was assessed using the Albumin-Bilirubin (ALBI) score and grade (13, 14). A total of 116 patients with 128 metastatic lesions were included in the analysis. This study was approved by the Institutional Review Board of the Samsung Medical Center (No. 2025-09-187). The requirement for informed consent was waived owing to the retrospective nature of the study.

### RT planning

The treating physicians determined the respiratory motion management approach, RT modality, and dose scheme based on tumor location, proximity to normal organs, and institutional availability, among other factors. Respiratory motion management was implemented using breath-hold (BH, n=20, 15.6%), gating (n=86, 67.2%), or regular free breathing (n=22, 17.2%) techniques. A simulation computed tomography (CT) scan was performed with a Vac-lok™ to minimize the potential movement of the body during simulation and RT. Four-dimensional (4D) CT scans with a contrast agent were acquired. Additional planning magnetic resonance imaging (MRI) using a contrast agent was optionally performed with the same position and devices in 90 cases (70.3%). All CT and MRI scans were taken with a 2.5-mm slice thickness.

The gross tumor volume (GTV)s were delineated on the six sets of serial CT images in the BH technique or using 4D-CT images covering the amplitude of the gating window or whole respiratory phase. A 0.3–0.5 cm margin was added to define the planning target volume (PTV). Median PTV volume was 35.5 cc (interquartile range [IQR], 20.1–72.2).

SBRT was delivered using X-ray RT (XRT) (39 lesions, 30.5%) and proton beam therapy (PBT) (89 lesions, 69.5%). XRT was delivered using intensity-modulated RT. PBT was delivered using a passive scattering (n=20, 15.6%) or pencil beam scanning method (n=69, 53.9%). For PBT, a relative biological effectiveness value of 1.1 was used. Baseline characteristics were generally similar between the two groups; however, the PBT group had a larger GTV volume (median, 22.2 vs. 4.6 cc, p=0.005) and slightly higher BED_10_ (median, 132.0 vs. 105.6 Gy, p=0.068). Image guidance was performed with daily cone-beam CT.

The most common prescription dose scheme was 60 GyE in 5 fractions (132.0 Gy BED_10_, 42.2%), followed by 70 GyE in 10 fractions (119.0 Gy BED_10_, 12.5%, **Supplementary Table 1**). The median prescription dose was 132.0 Gy BED_10_ (IQR, 112.5–132.0). RT planning aimed to ensure that 95% of the prescribed dose covered PTV and 100% covered GTV. Representative dose constraints are summarized in **Supplementary Table 2**.

### Assessments

After SBRT, the treatment response was evaluated according to the revised Response Evaluation Criteria in Solid Tumors (RECIST v1.1) (15). Radiation-induced liver disease (RILD) included classic and non-classic RILD. Classic RILD was defined as anicteric hepatomegaly, non-malignant ascites, or an elevation in alkaline phosphatase more than twice the upper limit of normal value. Non-classic RILD was defined as an elevation of liver transaminases more than 5 times the upper limit of normal level or a worsening of Child-Pugh score of ≥ 2 points. Other treatment-related toxicities were assessed using the National Cancer Institute Common Terminology Criteria for Adverse Events (CTCAE) version 5.0.

### Statistical analysis

The rates of FFLP and progression-free survival (PFS) were calculated from the start date of SBRT to the date of each event or death from any cause or the date of last follow-up, whichever occurred first. Overall survival (OS) was calculated from the start date of SBRT to death from any cause or last follow-up. Survival outcomes were estimated using the Kaplan-Meier method and compared using the log-rank test. Cox proportional hazards models were used for univariable and multivariable analyses.

To evaluate the impact of plan parameters on FFLP, dosimetric parameters including maximum dose (Dmax), minimum dose (Dmin), mean dose (Dmean), minimum dose to 2%, 95%, 98%, 99% of the PTV volume (D2%, D95%, D98%, D99%) were reviewed. To minimize the influence of dose scheme heterogeneity, all doses were converted to BED_10_ and included in the analysis. We calculated the Pearson correlation coefficient among dosimetric parameters that were significant in the univariable analysis. Given the high inter-parameter correlations, a null multivariable Cox proportional hazards model was initially built based on significant clinical factors only. Subsequently, separate multivariable models were constructed by adding each dosimetric parameter individually to the null model. The most discriminative multivariable model was selected based on the lowest Akaike information criterion (AIC). Prognostic performance was assessed using out-of-fold predictions from stratified 10-fold cross-validation, preserving event balance within folds. In each iteration, the model was trained on the remaining folds (Efron method for ties), and linear predictors for the held-out fold were generated and aggregated. Harrell’s C-index was calculated on the aggregated predictions to evaluate discrimination. The optimal cut-point for continuous variables was determined using the Maximal Log-Rank χ2 Test. To compare the distribution of treatment characteristics between the subgroups, a Chi-squared test or Fisher’s exact test for categorical variables, and the Wilcoxon rank-sum test for continuous variables were used.

The tumor control probability (TCP) was defined as the probability of tumor control at 12 months after SBRT. The X-axis of the TCP curve represents the prescribed dose or dosimetric parameter as BED_10_, and the Y-axis represents the probability of tumor control. The TCP for each bin was calculated using the Kaplan-Meier method. The quantification of the dose response to the tumor was estimated using a logistic model as follows: (D: total dose; γ: slope of the curve; and TCD50: dose that achieves a TCP of 50%). TCD50 and γ were estimated by a logit function. The 95% confidence intervals (CIs) were calculated using the probability density function of the normal distribution.


$$TCP=\frac{1}{{\left(1+\frac{TCD_{50}}{D}\right)}^{\frac{4}{\text{γ}}}}$$

Statistical significance was set at p<0.05. All statistical analyses were performed using the R software (version 4.4.3; R Foundation for Statistical Computing, Vienna, Austria; https://www.R-project.org/).

## Results

### Baseline and treatment characteristics

The baseline and treatment characteristics are summarized in **Table 1**. The median age was 61 years (IQR, 52–67), and 63.3% were male. At the time of SBRT, 101 (78.9%) cases were categorized as oligometastatic disease, while 27 (21.1%) were polymetastatic. According to ESTRO/EORTC classifications, 66 (51.6%) cases were repeat oligometastasis, 52 (40.6%) cases were induced oligometastasis, and 10 (7.8%) cases were *de novo* oligometastasis. The median carcinoembryonic antigen (CEA) level before SBRT was 6.1 ng/mL (IQR, 2.8–30.0), with elevated values observed in 72 (56.2%) cases. A total of 52 (40.6%) cases received concurrent systemic therapy during SBRT. The median number of pre-SBRT systemic therapy lines was 1 (IQR, 1–2), and 26 cases (20.3%) received 3 or more lines of systemic therapy.

**Table 1:** Baseline and treatment characteristics (N = 128).

Variables		
Age, years	(Continuous)	61 [52–67]
Sex	Male	81 (63.3%)
	Female	47 (36.7%)
TMB	Low	39 (30.5%)
	High	11 (8.6%)
	Unknown	78 (60.9%)
MSI	MSI-low	1 (0.8%)
	MSS	103 (80.5%)
	Unknown	24 (18.8%)
Tumor maximum diameter, cm	(Continuous)	2.8 [1.6–4.6]
ALBI score	(Continuous)	-5.8 [-6.0–5.5]
ALBI grade	Grade 1	128 (100%)
Metastasis extent at SBRT	Oligometastasis	101 (78.9%)
	Polymetastasis	27 (21.1%)
ESTRO/EORTC classification	*De novo*	10 (7.8%)
	Synchronous oligometastasis	2 (1.6%)
	Metachronous oligorecurrence	5 (3.9%)
	Metachronous oligoprogression	3 (2.3%)
	Repeated	66 (51.6%)
	Repeat oligorecurrence	26 (20.3%)
	Repeat oligopersistence	20 (15.6%)
	Repeat oligoprogression	20 (15.6%)
	Induced	52 (40.6%)
	Induced oligorecurrence	10 (7.8%)
	Induced oligopersistence	11 (8.6%)
	Induced oligoprogression	31 (24.2%)
Pre-SBRT CEA, ng/mL	(Continuous)	6.1 [2.8–30.0]
	Elevated (>5 ng/mL)	72 (56.2%)
Post-SBRT 1-month CEA, ng/mL	(Continuous)	5.1 [2.2–31.7]
	Elevated (>5 ng/mL)	32 (25.0%)
Pre-SBRT lines of systemic therapy	(Continuous)	1 [1–2]
	≥3	26 (20.3%)
Concurrent systemic therapy at SBRT	Yes	52 (40.6%)
Detailed information on SBRT		
SBRT modality	X-ray	39 (30.5%)
	Proton	89 (69.5%)
PTV, cc	(Continuous)	35.5 [20.1–72.2]
Prescription dose, Gy BED_10_	(Continuous)	132.0 [112.5–132.0]
PTV Dmax, Gy BED_10_	(Continuous)	139.1 [117.8–146.4]
PTV Dmin, Gy BED_10_	(Continuous)	88.6 [54.8–116.6]
PTV Dmean, Gy BED_10_	(Continuous)	126.3 [110.9–134.7]
PTV D2%, Gy BED_10_	(Continuous)	137.0 [115.0–141.9]
PTV D95%, Gy BED_10_	(Continuous)	111.8 [95.4–130.3]
PTV D98%, Gy BED_10_	(Continuous)	107.6 [84.0–127.4]
PTV D99%, Gy BED_10_	(Continuous)	103.8 [78.3–125.9]

Values are presented as the number of patients (%) or the median [interquartile range]. TMB, tumor mutation burden; MSI, microsatellite instability; MSS, microsatellite stable; ALBI, Albumin-Bilirubin; SBRT, stereotactic body radiation therapy; ESTRO, the European Society for Radiotherapy and Oncology; EORTC, European Organization for Research and Treatment of Cancer; CEA, carcinoembryonic antigen; PTV, planning target volume; BED_10_, biologically effective dose with α/β of 10; Dmax, maximum dose; Dmin, minimum dose; Dmean, mean dose; Dx%, minimum dose to x% of the PTV.

### Prescription dose and DVH metrics

**Figure 1:**
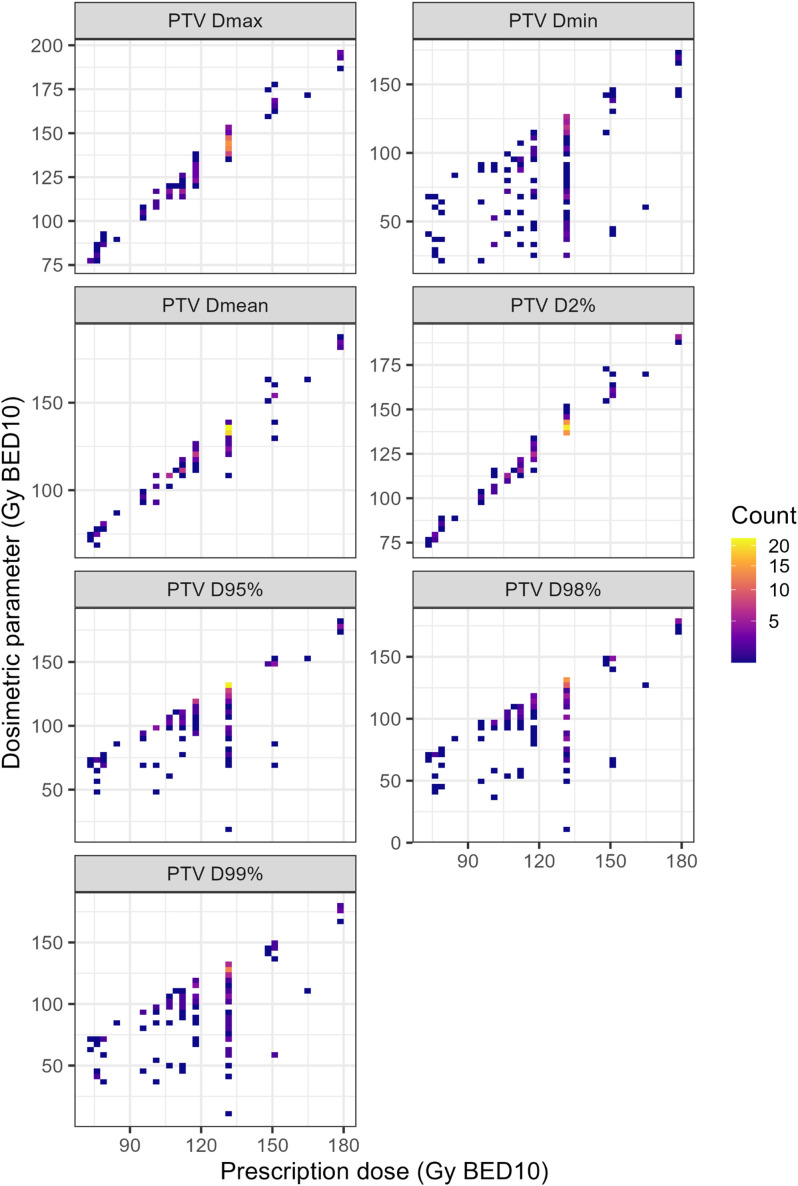
Two−dimensional frequency maps between prescription dose and each dosimetric metric. Doses are calculated as biologically effective dose (BED) with α/β of 10.

A Pearson correlation matrix demonstrated strong correlations between prescription dose and most of the dosimetric parameters (**Supplementary Figure 1**). However, **Figure 1** illustrates substantial inter-case variability in individual DVH parameters (Dmax, Dmean, Dmin, D2%, D95%, D98%, D99%), even among cases treated with identical prescription dose. This variability indicates that the nominal prescription dose does not fully represent the actual dose distribution delivered to the tumor, underscoring the potential relevance of DVH-based plan quality parameters.

### Oncologic outcome and prognostic factors

**Figure 2:**
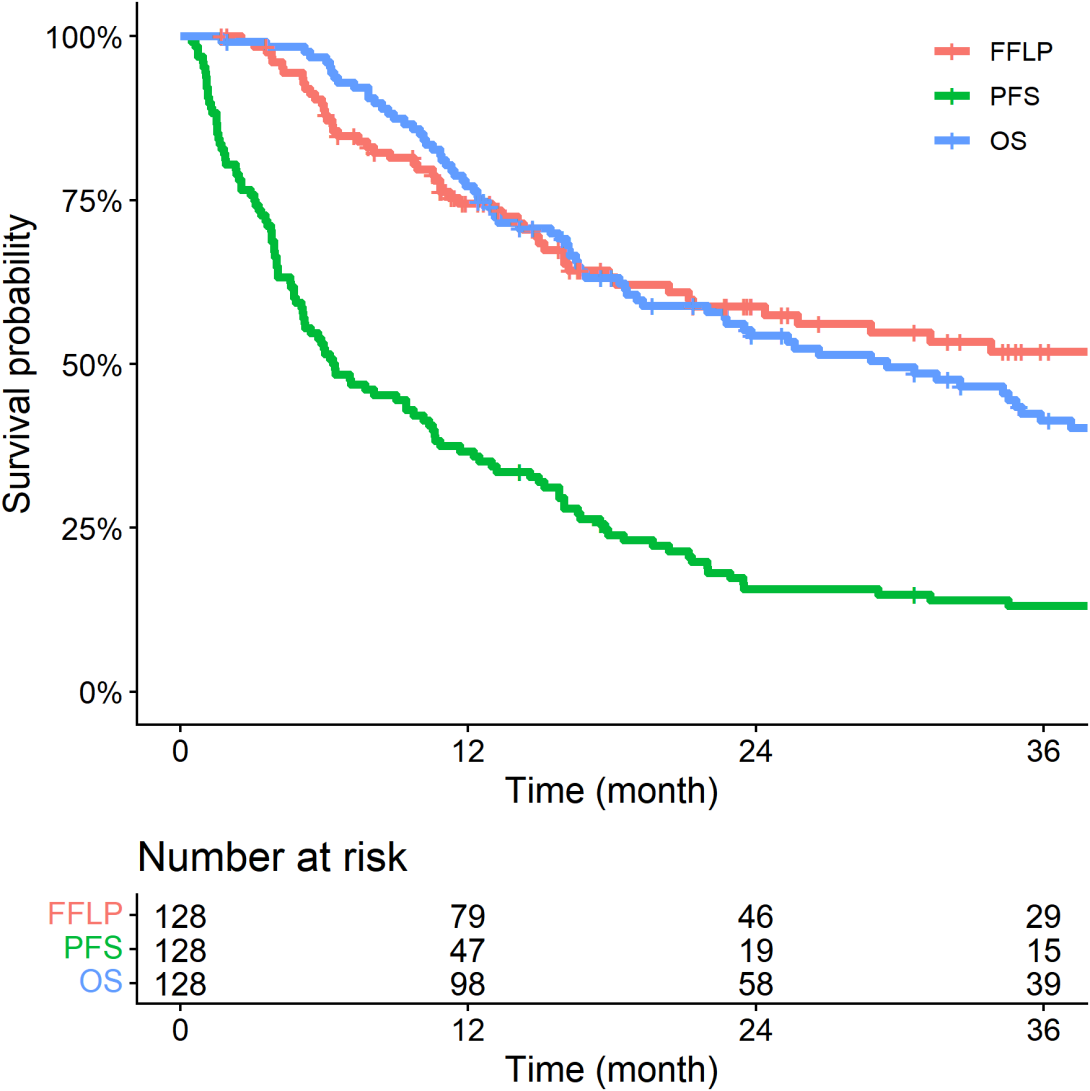
Kaplan-Meier survival curves of freedom from local progression (FFLP), progression-free survival (PFS), and overall survival (OS) for the entire cohort.

With a median follow-up of 22.3 months (IQR, 12.4–43.4), the 1- and 2-year FFLP rates were 74.5% and 58.8%, respectively. The corresponding PFS rates were 37.6% and 16.6%, and the OS rates were 77.2% and 54.3%, respectively (**Figure 2**).

In the univariable analyses for FFLP, polymetastatic disease, a greater number of prior systemic therapy lines, higher pre-SBRT CEA levels, higher post-SBRT 1-month CEA levels, and larger PTV were associated with inferior outcomes. Among dosimetric variables, higher prescription dose, and all evaluated DVH parameters (Dmax, Dmin, Dmean, D2%, D95%, D98%, and D99% of PTV) were significantly associated with improved FFLP (p<0.01 for all) (**Table 2**).

**Table 2:** Prognostic factors for freedom from local progression.

		Univariable analysis		Multivariable model 1		Multivariable model 2	
Variables	(ref. vs.)	HR (95% CI)	P-value	HR (95% CI)	P-value	HR (95% CI)	P-value
Clinical factors							
Age	(Continuous)	0.99 (0.96–1.01)	0.180				
Sex	(Male vs. Female)	1.05 (0.59–1.86)	0.866				
TMB	(Low/Unknown vs. High)	1.00 (0.73–1.35)	0.973				
Primary treatment	(Curative resection vs None)	0.49 (0.18–1.35)	0.166				
Metastasis extent at SBRT	(Oligometastasis vs. Polymetastasis)	3.35 (1.82–6.15)	<0.001	2.72 (1.33–5.57)	0.006	3.31 (1.79–6.12)	<0.001
ESTRO/EORTC Classification	(*De novo* vs Repeat)	2.45 (0.58–10.3)	0.221				
	(*De novo* vs. Induced)	2.40 (0.56–10.3)	0.236				
Pre-SBRT lines of systemic therapy	(Continuous)	1.23 (1.05–1.43)	0.010	1.15 (0.94–1.40)	0.200		
Concurrent chemotherapy at SBRT	(No vs. Yes)	0.85 (0.48–1.50)	0.584				
Pre-SBRT CEA, per 10ng/mL	(Continuous)	1.02 (1.01–1.03)	0.001	1.02 (0.99–1.03)	0.300		
Post-SBRT 1-month CEA, per 10ng/mL	(Continuous)	1.00 (1.00–1.00)	0.006	1.00 (0.98–1.00)	0.600		
SBRT modality	(X-ray vs. Proton)	1.05 (0.58–1.92)	0.862				
Respiratory control	(Regular free breathing vs. BH)	1.69 (0.60–4.77)	0.318				
	(Regular free breathing vs. Gating)	1.68 (0.71–4.00)	0.238				
PTV, per 10cc	(Continuous)	1.04 (1.02–1.06)	<0.001	1.01 (0.98–1.06)	0.400		
Dosimetric parameters							
Prescription dose, per 10 Gy BED_10_	(Continuous)	0.81 (0.71–0.93)	0.002				
Dmax, per 10 Gy BED_10_	(Continuous)	0.85 (0.75–0.95)	0.006				
Dmin, per 10 Gy BED_10_	(Continuous)	0.89 (0.83–0.96)	0.002			0.89 (0.83–0.96)	0.002
Dmean, per 10 Gy BED_10_	(Continuous)	0.82 (0.72–0.93)	0.002				
D2%, per 10 Gy BED_10_	(Continuous)	0.84 (0.74–0.95)	0.005				
D95%, per 10 Gy BED_10_	(Continuous)	0.87 (0.80–0.95)	0.002				
D98%, per 10 Gy BED_10_	(Continuous)	0.88 (0.82–0.96)	0.002				
D99%, per 10 Gy BED_10_	(Continuous)	0.89 (0.82–0.96)	0.002				

Multivariable model 1 (baseline null model) includes significant clinical factors only. Multivariable model 2 is the final model, derived by incorporating the dosimetric parameter into multivariable model 1 based on the final model selection process. HR, hazard ratio; CI, confidence interval; TMB, tumor mutation burden; SBRT, stereotactic body radiation therapy; ESTRO, the European Society for Radiotherapy and Oncology; EORTC, European Organization for Research and Treatment of Cancer; CEA, carcinoembryonic antigen; BH, breath-hold; PTV, planning target volume; BED_10_, biologically effective dose with α/β of 10; Dmax, maximum dose; Dmin, minimum dose; Dmean, mean dose; Dx%, minimum dose to x% of the PTV.

For multivariable analysis, a two-step modeling approach was employed. First, a baseline null multivariable model incorporating only clinical factors identified polymetastatic disease at SBRT as the only independent predictor of FFLP (hazard ratio [HR] 2.72; 95% CI 1.33–5.57; p=0.006, **Table 2**). Second, each dosimetric parameter was added individually to the baseline null multivariable model. The model incorporating PTV Dmin demonstrated the best performance, yielding the lowest AIC and the highest out-of-fold Harrell’s C-index (C = 0.673, **Supplementary Table 3**). Accordingly, the final multivariable Cox model included both clinical and dosimetric factors. In this model, polymetastasis at SBRT (HR 3.31; 95% CI 1.79–6.12; p<0.001) and PTV Dmin (HR 0.89; 95% CI 0.83–0.96; p=0.002) remained independently associated with FFLP outcomes (**Table 2**).

**Figure 3:**
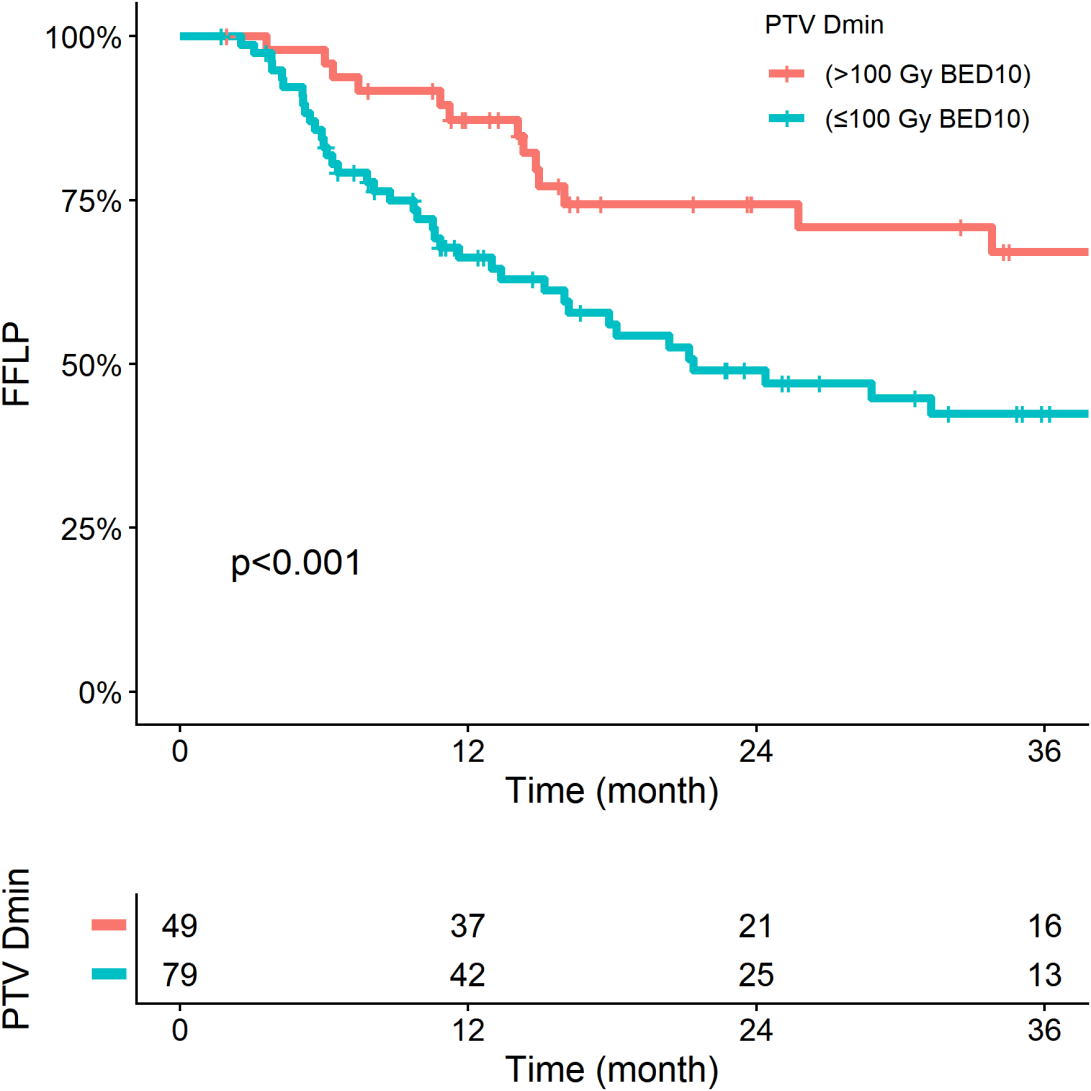
Freedom from local progression (FFLP) according to minimum dose of planning target volume (PTV Dmin). BED_10_, biologically effective dose with α/β of 10.

Based on the optimal cut-point for PTV Dmin (100 Gy BED_10_) determined by the Maximal Log-Rank χ2 Test, the low-PTV Dmin group (n=79, 61.7%) showed worse FFLP than the high-PTV Dmin group (n=49, 38.3%) (1-year: 64.2% vs. 87.1%; 2-year: 45.6% vs. 75.6%, p<0.001, **Figure 3**). **Supplementary Figure 2** presents the distribution of prescription doses stratified by PTV Dmin category and demonstrates that even among cases receiving the same nominal prescription dose, the achieved PTV Dmin varied substantially.

Regarding PFS, tumor mutational burden, metastasis extent at SBRT, and post-SBRT 1-month CEA levels were identified as significant prognostic factors. For OS, metastasis extent at SBRT remained the only significant prognostic factor after multivariable analysis (**Supplementary Table 4**).

### TCP modeling for FFLP

**Figure 4:**
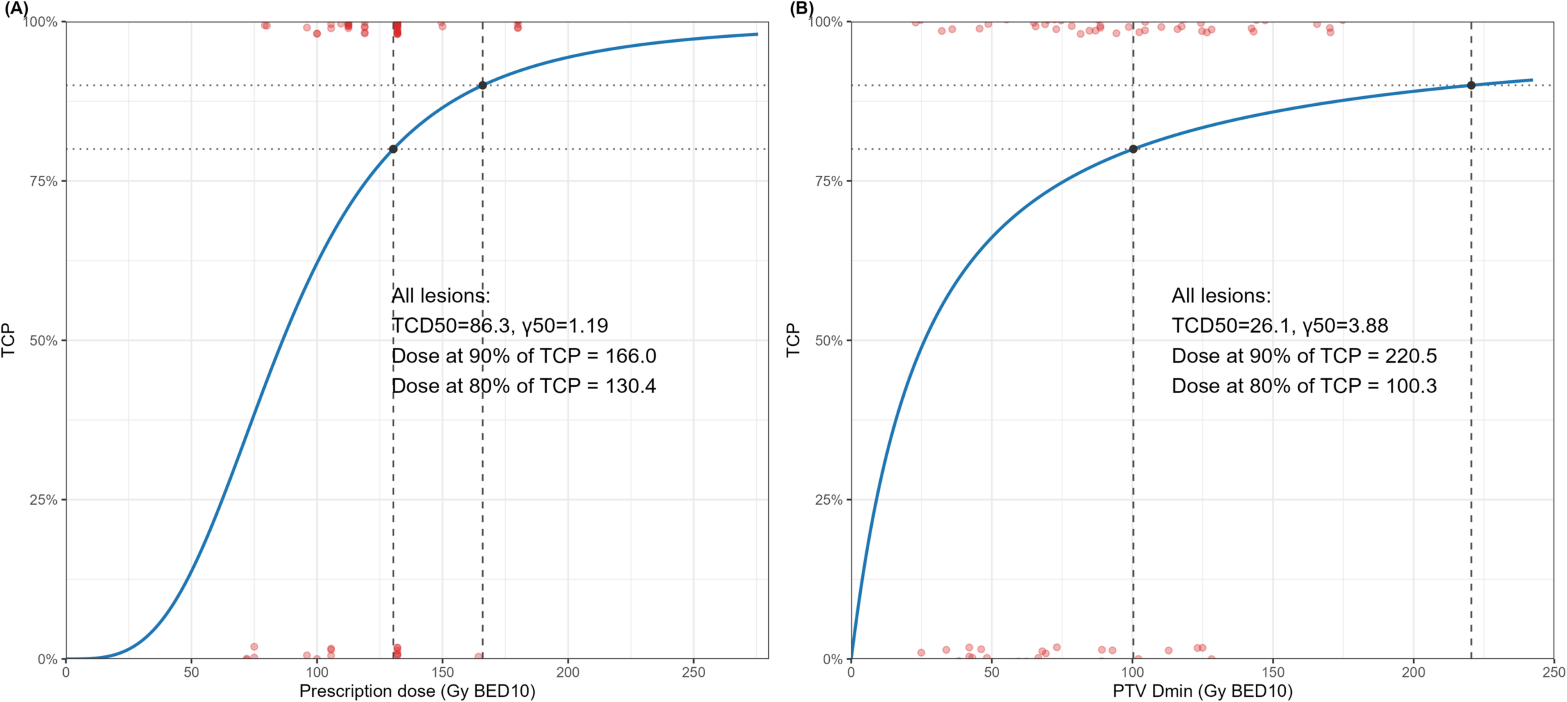
Tumor control probability (TCP) curve of dosimetric parameter versus 1-year freedom from local progression; **(A)** Prescription dose, **(B)** PTV Dmin. PTV, planning target volume; Dmin, minimum dose; BED_10_, biologically effective dose with α/β of 10; TCD50, tumor dose at which 50% of TCP is expected; γ50, the slope of the curve at TCD50.

All dosimetric parameters (prescription dose, Dmax, Dmean, Dmin, D2%, D95%, D98%, and D99%) demonstrated a clear dose-response relationship (**Figure 4**, **Supplementary Figure 3**). For prescription dose, the TCP model estimated a γ_50_ of 1.19 and a TCD_50_ of 86.3 Gy BED_10_ (95% CI, 66.7-106.0). The doses required to achieve 90% and 80% 1-year TCP were 166.0 Gy BED_10_ (95% CI, 139.1-331.2) and 130.4 Gy BED_10_ (95% CI, 114.5-171.2), respectively (**Figure 4**). For PTV Dmin, the TCP model yielded a γ_50_ of 3.88 and a TCD_50_ of 26.1 Gy BED_10_ (95% CI, 3.9-48.3), with corresponding doses of 220.5 Gy BED_10_ (95% CI, 118.0-4925.6) and 100.3 Gy BED_10_ (95% CI, 64.4-320.3) required to achieve 90% and 80% TCP, respectively (**Figure 4**).

### Treatment-related toxicities

Overall, grade 2 or higher acute toxicities occurred in six cases (4.7%), which included Grade 2 dermatitis (n=1, 0.8%), Grade 2 esophagitis (n=1, 0.8%), Grade 2 nausea and vomiting (n=2, 1.6%), and Grade 2 abdominal pain (n=3, 2.3%). As for late toxicity, only one case of grade 2 pneumonitis (0.8%) was observed. The RILD incidence rate was 14.1% (n=18). Overall, there was no significant difference in prescription dose (median BED_10_, 132.0 Gy [IQR, 75.1–132.0] vs. 132.0 Gy [IQR, 112.5–132.0], p=0.510) between cases with and without RILD. Although cases treated with PBT received a higher BED_10_ (median, 132.0 Gy vs. 105.6 Gy, p=0.068), they exhibited numerically lower RILD rates compared with those treated with XRT (11.2% vs. 20.5%, p=0.266).

## Discussion

In this study, we evaluated FFLP outcomes and the impact of actual dosimetric plan quality in SBRT for CLM. Among all dosimetric metrics, PTV Dmin emerged as the strongest predictor of FFLP, outperforming the nominal prescription dose itself. These findings indicate that the minimum dose delivered within the target – reflecting the “cold spot” – may play a more important role in determining local control after liver SBRT than the nominal prescription dose.

Although the prescription dose reflects the intended treatment goal, the actual delivered dose within the PTV varied substantially in our cohort, even among cases intended to receive identical prescriptions (**Figure 1**, **Supplementary Figure 2**). Such variability likely reflects the influence of organ-at-risk constraints, motion management, or planning strategies. Previous studies of SBRT have similarly reported that delivered dose heterogeneity, rather than the nominal prescription dose, better predicts clinical outcomes (16–23). Several studies have shown that higher PTV Dmax, reflecting high dose (“hot spots”) within the PTV, correlates with improved FFLP and survival outcomes (16, 18, 19, 21). In the context of liver-directed SBRT, Kang et al. similarly reported that Dmax was a significant predictor of local control in colorectal oligometastases treated with SBRT, supporting the relevance of dose intensity metrics beyond nominal prescription dose (22). Furthermore, a radiobiological modeling study also suggested that inhomogeneous SBRT plans with higher intratumoral maximum doses may enhance TCP compared with homogeneous plans (23). Meanwhile, a lung SBRT study by Zhao et al. demonstrated that when PTV D95% fell below a threshold dose (≤ 86 Gy BED_10_), the risk of local progression increased significantly (17). Similarly, in spine SBRT, a retrospective study of 522 lesions identified GTV Dmin as the strongest predictor of local failure, with minimum dose ≥ 14–15 Gy associated with lower recurrence rates (20). Consistent with these findings, our data demonstrated that PTV Dmin, not the prescription dose, was the most meaningful predictor of FFLP, suggesting that “cold spots” within the target may play a critical role in determining local failure. Furthermore, the markedly steeper γ_50_ observed in the TCP model for PTV Dmin compared with prescription dose (3.88 vs. 1.19) supports this interpretation, indicating that small variations in minimum delivered dose translate into large differences in TCP.

In the current study, inferior FFLP rates (1-year 74.5%, 2-year 58.8%) may reflect the inherent radioresistance of colorectal cancer. Prior studies consistently reported lower dose responsiveness in colorectal metastases than in other primaries, with substantially higher BED required to achieve durable local control (8, 24–28). Chen et al. found that 2-year FFLP was only 42% when < 96 Gy BED_10_ was delivered, and that achieving a TCP >70% required estimated doses exceeding 185–250 Gy BED_10_ (9). In contrast, liver metastases from breast cancer exhibit higher control rates at similar BED levels, further reinforcing the relative radioresistance of CLM (29). Our TCP modeling aligns with these observations, demonstrating that a dose of approximately 130–166 Gy BED_10_ is required to achieve 80–90% predicted 1-year FFLP. This substantial dose requirement underscores the importance of achieving adequate dose coverage within the PTV. Underdosed subvolumes (“cold spots”) may harbor more resistant or hypoxic tumor regions, thereby increasing the risk of local failure. Our finding that PTV Dmin is the strongest predictor of FFLP supports this biologic rationale of radioresistance in CLM.

However, dose escalation in liver SBRT is fundamentally constrained by hepatic tolerance. The HyTEC guidelines highlight the strong dose-volume sensitivity of the liver and the importance of adhering to strict volumetric limits (30, 31). In this context, the physical characteristics of PBT allow for superior normal-liver sparing, potentially enabling safe dose escalation in liver SBRT (32–34). In our cohort, the prescribed BED_10_ was slightly higher in the PBT group compared to the XRT group (median 132.0 Gy BED_10_ vs. 105.6 Gy BED_10,_ p=0.068). Despite this higher target dose, PBT showed a lower tendency for RILD compared with XRT (11.2% vs. 20.5%, p=0.266). This reduction in hepatic toxicity supports the potential role of PBT in clinical scenarios where dose escalation is required for CLM.

This study has some limitations. First, its retrospective design and the heterogeneous clinical courses, including disease extent, systemic therapy, and SBRT dose schemes, might limit the ability to isolate SBRT-specific effects. Prospective validation with standardized treatment protocols is warranted to establish PTV Dmin-based planning objectives as an important component in SBRT planning for CLM. Second, the modest C-index of the final model (0.673) indicates room for improvement, potentially through integration of radiomics or biologic tumor characteristics. Third, the wide 95% CI in the TCP model for PTV Dmin may limit its direct application as a prescriptive threshold; therefore, the TCP curve should be interpreted as an exploratory characterization of the dose–response relationship, pending prospective validation. Despite these limitations, the study also has strengths. Previous studies on liver-directed RT for CLM largely focused on prescription dose (9, 25, 35–39) with few studies evaluating DVH-based plan quality metrics. To our knowledge, no previous report has identified PTV Dmin as the most discriminative dosimetric predictor of FFLP in CLM. Our findings suggest that PTV Dmin may serve as a clinically meaningful and actionable dose constraint for future liver SBRT planning and clinical trial design, addressing a gap in the current literature.

In conclusion, the minimum target dose emerged as the most important dosimetric predictor of FFLP in SBRT for CLM, surpassing the predictive value of the nominal prescription dose. These findings highlight the clinical relevance of cold-spot avoidance and support incorporating adequate PTV Dmin as a planning objective in liver SBRT. Future work should further refine Dmin-based constraints and evaluate their integration with advanced SBRT planning.

## Data Availability

The raw data supporting the conclusions of this article will be made available by the authors, without undue reservation.
