# Supplementary material for: Cold-spot driven local failure after stereotactic body radiation therapy for colorectal liver metastases
Source: Front Oncol. 2026 Apr 17;16:1800905. doi: 10.3389/fonc.2026.1800905 (PMC13132765; doi:10.3389/fonc.2026.1800905)
Supplement: Supplementary file 1 [file DataSheet1.docx]

**Supplementary Figure 1. A Pearson correlation matrix showing associations between dosimetric parameters.** Abbreviations: PTV, planning target volume; Dmax, maximum dose; Dmin, minimum dose; Dmean, mean dose; Dx%, minimum dose to x% of the PTV; Corr, Pearson correlation coefficient.


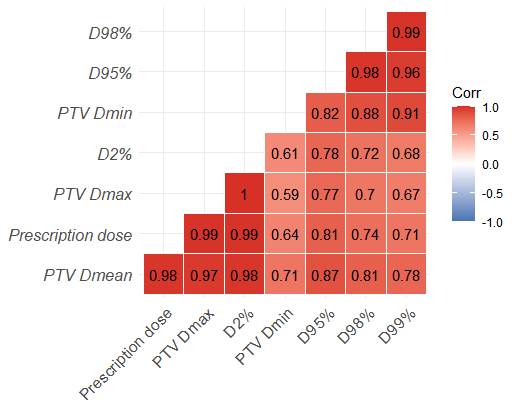


**Supplementary Figure 2. Prescription dose distribution by minimum dose (Dmin) of planning target volume (PTV) group based on optimal cut-point (high >100 Gy BED_10_ vs. low ≤100 Gy BED_10_). The thick horizontal line indicates the median, boxes represent the first and third quartiles, and the whiskers extend to the observed minimum and maximum.**


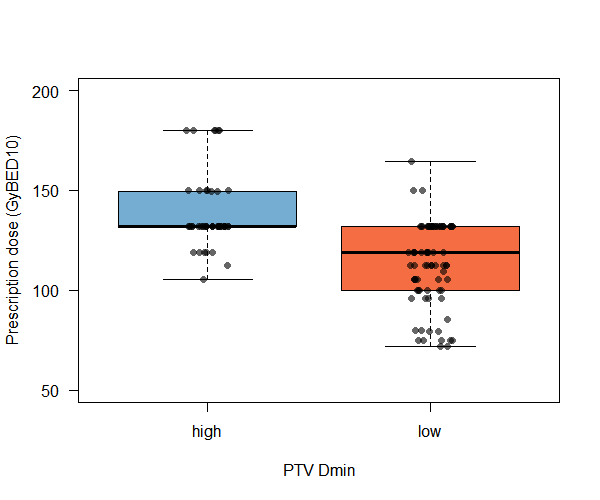


**Supplementary Figure 3. Tumor control probability (TCP) curve of dosimetric parameter versus 1-year freedom from local progression; (A) Dmax, (B) Dmean, (C) D2%, (D) D95%, (E) D98%, (F) D99% of PTV.** Abbreviations: PTV, planning target volume; Dmax, maximum dose; Dmean, mean dose; Dx%, minimum dose to x% of the PTV; BED_10_, biologically effective dose with α/β of 10; TCD50, tumor dose at which 50% of TCP is expected; γ50, the slope of the curve at TCD50.

**
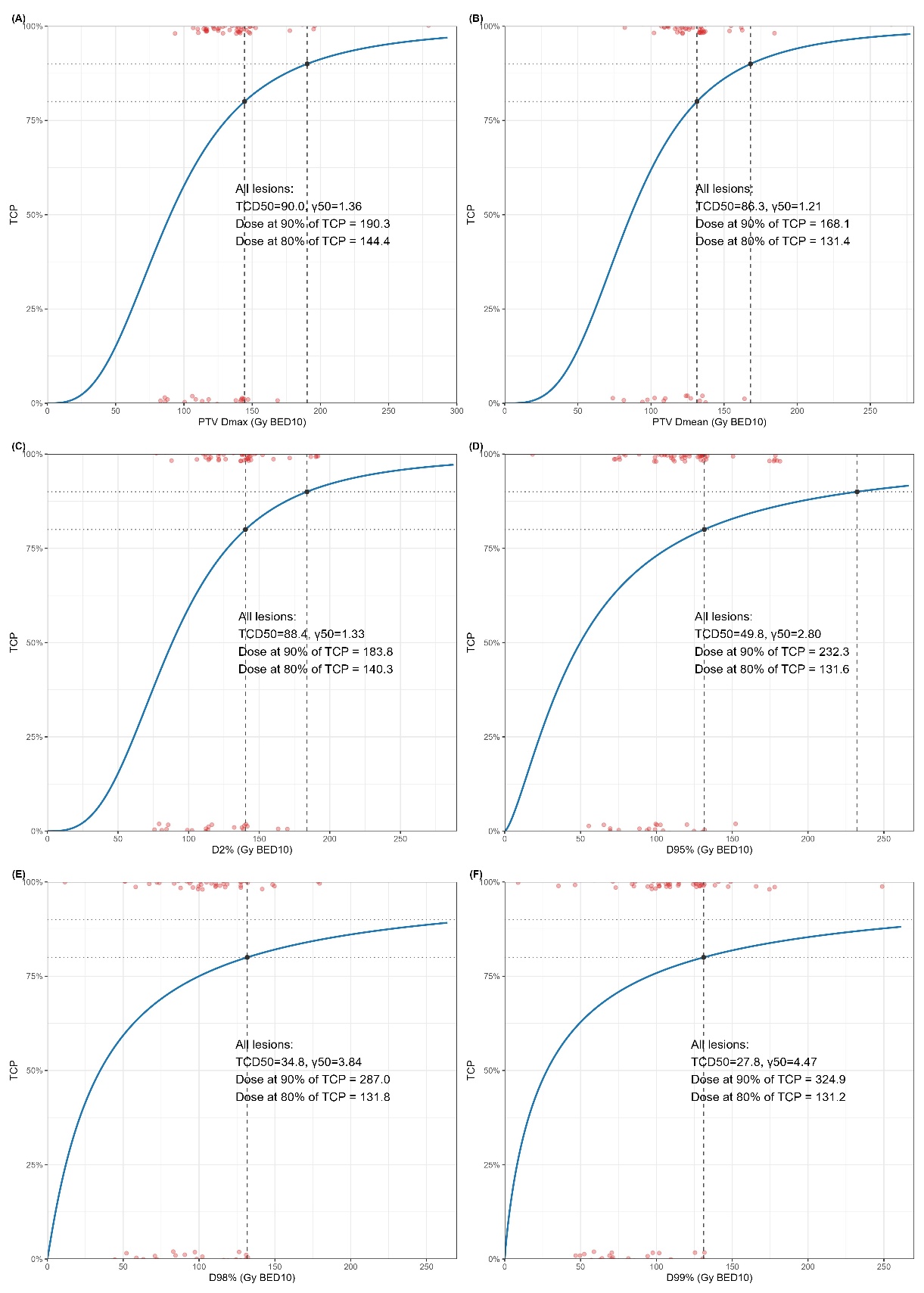
**

**Supplementary Table 1. Details of prescription dose & fractionation (N=128)**

| Dose scheme | BED_10_ (Gy) | N (%) |
| --- | --- | --- |
| 40 GyE/5 fractions | 72.0 | 2 (1.6) |
| 50 GyE/10 fractions | 75.0 | 4 (3.1) |
| 45.5 GyE/7 fractions | 75.1 | 1 (0.8) |
| 36 GyE/3 fractions | 79.2 | 2 (1.6) |
| 40 GyE/4 fractions | 80.0 | 2 (1.6) |
| 45 GyE/5 fractions | 85.5 | 1 (0.8) |
| 60 GyE/10 fractions | 96.0 | 4 (3.1) |
| 50 GyE/5 fractions | 100.0 | 6 (4.7) |
| 48 GyE/4 fractions | 105.6 | 7 (5.5) |
| 66 GyE/10 fractions | 109.6 | 1 (0.8) |
| 75 GyE/15 fractions | 112.5 | 6 (4.7) |
| 45 GyE/3 fractions | 112.5 | 5 (3.9) |
| 70 GyE/10 fractions | 119.0 | 16 (12.5) |
| 60 GyE/5 fractions | 132.0 | 54 (42.2) |
| 65 GyE/5 fractions | 149.5 | 2 (1.6) |
| 60 GyE/4 fractions | 150.0 | 7 (5.5) |
| 99 GyE/15 fractions | 164.3 | 1 (0.8) |
| 60 GyE/3 fractions | 180.0 | 6 (4.7) |
| 75 GyE/3 fractions | 262.5 | 1 (0.8) |

Abbreviations: BED_10_, biologically effective dose with α/β of 10

**Supplementary Table 2. Normal organ dose constraints**

| Organ at risks | Volume | 10 fractions | 5 fractions |
| --- | --- | --- | --- |
| Esophagus max | <0.5 cc | 40 Gy | 32 Gy |
| Stomach max (α/β = 3) | <0.5 cc | 35 Gy | 30 Gy |
|  | <5 cc | 30 Gy | 25 Gy |
| Duodenum max (α/β = 3) | <0.5 cc | 35 Gy | 30 Gy |
|  | <5 cc | 30 Gy | 25 Gy |
| Small bowel max (α/β = 3) | <0.5 cc | 35 Gy | 30 Gy |
|  | <5 cc | 30 Gy | 25 Gy |
| Large bowel max | <0.5 cc | 40 Gy | 32 Gy |
| Heart/Pericardium | <30 cc | 39 Gy | 30 Gy |
| Great vessel max | <0.5 cc | 60 Gy | 30 Gy |
| Cord + 5 mm max | <0.5 cc | 33 Gy | 25 Gy |
| Kidneys (α/β = 10) | Bilateral mean dose | 11 Gy | 10 Gy |
| Skin max (α/β = 10) | <0.5 cc | 38 Gy | 32 Gy |
| Chest wall max | <0.5 cc | 60 Gy | 50 Gy |
| Gallbladder max | <0.5 cc | 60 Gy | 55 Gy |
| Common bile duct max | <0.5 cc | 60 Gy | 50 Gy |

**Supplementary Table 3. Competing multivariable Cox regression model incorporating dosimetric parameters for freedom from local progression**

| Variables | AIC | C-index |
| --- | --- | --- |
| Metastasis extent | 447 | 0.598 |
| Metastasis extent + prescription dose | 442 | 0.635 |
| Metastasis extent + PTV Dmax | 444 | 0.625 |
| Metastasis extent + PTV Dmin | **439** | **0.673** |
| Metastasis extent + PTV Dmean | 442 | 0.641 |
| Metastasis extent + PTV D2% | 443 | 0.630 |
| Metastasis extent + PTV D95% | 441 | 0.658 |
| Metastasis extent + PTV D98% | 441 | 0.662 |
| Metastasis extent + PTV D99% | 440 | 0.668 |

Abbreviations: AIC, Akaike information criterion; PTV, planning target volume; Dmax, maximum dose; Dmin, minimum dose; Dmean, mean dose; Dx% = minimum dose to x% of PTV; C-index, out-of-fold Harrell’s C-index. All doses were converted to biologically effective dose using an α/β ratio of 10.

**Supplementary Table 4.** **Prognostic factors for progression-free survival and overall survival**

| **Progression-free survival** |  | **Univariable** | | **Multivariable** | |
| --- | --- | --- | --- | --- | --- |
| Variables | (ref. vs.) | HR (95% CI) | P-value | HR (95% CI) | P-value |
| **Age**, years | (Continuous) | 0.99 (0.97-1.00) | 0.076 |  |  |
| **Sex** | (Male vs. Female) | 1.24 (0.85-1.81) | 0.272 |  |  |
| **TMB** | (Low/Unknown vs. High) | 0.77 (0.63-0.94) | 0.011 | 0.74 (0.57-0.96) | 0.026 |
| **Primary treatment** | (Surgery vs None) | 1.09 (0.66-1.81) | 0.732 |  |  |
| **Metastasis extent at SBRT** | (Oligometastasis vs. Polymetastasis) | 3.00 (1.90-4.73) | <0.001 | 2.47 (1.47-4.15) | <0.001 |
| **ESTRO/EORTC classification** | (De novo vs Repeat) | 1.64 (0.75-3.61) | 0.216 |  |  |
|  | (De novo vs. Induced) | 1.98 (0.89-4.40) | 0.095 |  |  |
| **Pre-SBRT lines of systemic therapy** | (Continuous) | 1.15 (1.02-1.31) | 0.022 | 1.16 (1.00-1.36) | 0.051 |
| **Concurrent chemotherapy** | (No vs. Yes) | 1.56 (1.07-2.26) | 0.021 | 1.03 (0.64-1.67) | 0.999 |
| **Pre-SBRT CEA,** per 10ng/mL | (Continuous) | 1.02 (1.01-1.03) | <0.001 | 0.99 (0.97-1.02) | 0.600 |
| **Post-SBRT 1-month CEA,** per 10ng/mL | (Continuous) | 1.02 (1.01-1.03) | 0.002 | 1.02 (1.00-1.03) | 0.048 |
| **SBRT modality** | (X-ray vs. Proton) | 1.16 (0.78-1.73) | 0.457 |  |  |
| **PTV**, per 10cc | (Continuous) | 1.01 (0.99-1.03) | 0.320 |  |  |
| **Dmin**, per 10 Gy BED_10_ | (Continuous) | 0.97 (0.92-1.01) | 0.174 |  |  |
| **Overall survival** |  | **Univariable** | | **Multivariable** | |
| Variables | (ref. vs.) | HR (95% CI) | P-value | HR (95% CI) | P-value |
| **Age**, years | (Continuous) | 1.00 (0.98-1.02) | 0.877 |  |  |
| **Sex** | (Male vs. Female) | 1.41 (0.91-2.18) | 0.122 |  |  |
| **TMB** | (Low/Unknown vs. High) | 0.84 (0.66-1.06) | 0.140 |  |  |
| **Primary treatment** | (Surgery vs None) | 1.54 (0.86-2.75) | 0.146 |  |  |
| **Metastasis extent at SBRT** | (Oligometastasis vs. Polymetastasis) | 4.11 (2.50-6.75) | <0.001 | 3.82 (2.20-6.63) | <0.001 |
| **ESTRO/EORTC classification** | (De novo vs Repeat) | 0.98 (0.42-2.0) | 0.958 |  |  |
|  | (De novo vs. Induced) | 1.20 (0.50-2.86) | 0.684 |  |  |
| **Pre-SBRT systemic therapy** | (No vs. Yes) | 1.82 (0.57-5.77) | 0.309 |  |  |
| **No. systemic therapy line** | (Continuous) | 1.05 (0.92-1.21) | 0.450 |  |  |
| **Concurrent chemotherapy** | (No vs. Yes) | 1.48 (0.95-2.30) | 0.079 |  |  |
| **Pre-SBRT CEA,** per 10ng/mL | (Continuous) | 1.01 (1.00-1.02) | 0.003 | 1.01 (0.98-1.04) | 0.600 |
| **Post-SBRT 1-month CEA,** per 10ng/mL | (Continuous) | 1.01 (1.00-1.01) | 0.005 | 1.00 (0.98-1.01) | 0.800 |
| **SBRT modality** | (X-ray vs. Proton) | 0.84 (0.53-1.33) | 0.468 |  |  |
| **PTV**, per 10cc | (Continuous) | 1.03 (1.01-1.05) | 0.003 | 1.00 (0.98-1.03) | 0.800 |
| **Dmin**, per 10 Gy BED_10_ | (Continuous) | 0.94 (0.88-0.99) | 0.025 | 0.95 (0.89-1.02) | 0.140 |

Abbreviations: HR, hazard ratio; CI, confidence interval; TMB, tumor mutation burden; SBRT, stereotactic body radiation therapy; ESTRO, the European Society for Radiotherapy and Oncology; EORTC, European Organization for Research and Treatment of Cancer; CEA, carcinoembryonic antigen; PTV, planning target volume; Dmin, minimum dose; BED_10_, biologically effective dose using an α/β ratio of 10.
